# A survey of communication supports in Grade R classrooms in the Western Cape, South Africa

**DOI:** 10.4102/sajcd.v69i1.871

**Published:** 2022-10-27

**Authors:** Harsha Kathard, Prianka Parusnath, Lehana Thabane, Mershen Pillay, Zheng Jing Hu, Jane le Roux, Michal Harty, Shelley O’Carroll, Rizwana B. Mallick

**Affiliations:** 1 Department of Health and Rehabilitation Sciences, Faculty of Health Sciences, University of Cape Town, Cape Town, South Africa; 2 Department of Communication Sciences and Disorders, Faculty of Health Sciences, University of Cape Town, Cape Town, South Africa; 3 Department of Health Research Methods, Evidence and Impact, Faculty of Health Sciences, McMaster University, Hamilton, Canada; 4 Department of Speech and Language Therapy, Institute of Education, Massey University, Auckland, New Zealand; 5 Department of Speech-Language Pathology, Faculty of Health Sciences, University of KwaZulu-Natal, Durban, South Africa; 6 Wordworks, Cape Town, South Africa; 7 Division of Communication Sciences and Disorders, Faculty of Health Sciences, University of Cape Town, Cape Town, South Africa

**Keywords:** communication, pre-school, literacy, language, Grade R

## Abstract

**Background:**

Early classroom communication supports are critical in influencing oral language development and emergent literacy skills. It is both support from peers and adults, as well as the environment that impacts the quality and efficacy of language learning. Schools in particular play a key role in communication development, which will be further explored in this article.

**Objectives:**

To describe how communication was being supported in the classroom by teachers in the areas of language-learning environment (LLE), language-learning interactions (LLIs) and language-learning opportunities (LLOs) to focus on classroom communication supports to strengthen literacy outcomes for Grade R learners.

**Method:**

A cross-sectional descriptive survey of classrooms was conducted using a structured observation method in 136 classrooms. The Communication Supporting Classroom Observation Tool was used to make 223 observations across classrooms of LLE (19 items), LLI (20 items) and LLO (5 items). A descriptive analysis of frequency of occurrence of items was conducted for each domain.

**Results:**

Language-learning environment had more frequently occurring observations, with 12 out of 19 items being observed with a frequency of occurrence greater than 65%, indicating that the environment had adequate basic resources (e.g. good light). Language-learning interactions scores indicated that 3 out of 20 items were observed frequently, while 17 out of 20 items were observed less frequently (56.5%), including interactive strategies (e.g. turn-taking). Language Learning Opportunities scores indicated that teacher-led behaviours (e.g. including children in small group activities) were infrequently observed on all items.

**Conclusion:**

While the physical environment was generally supportive, the behaviours in the interactive domains of LLI and LLO were observed less frequently. There is potential for speech–language therapists to work collaboratively with teachers to develop communication supporting classrooms as a critical primary-level intervention for language-literacy learning.

## Introduction

There are two key components of education, viz. communication and language. Communication, as speech–language therapists have come to understand it, refers to the sending and receiving of messages with shared understanding, while language refers to the complex system of symbols that are used in a variety of modes for communication and thought (Huff & Christensen, 2018). Without meaningful communication, learning cannot happen, and without language, shared meaning and complex thoughts such as an understanding of literacy cannot develop. Language is the golden thread that underlies listening, speaking, writing and reading and is essential to literacy learning (Sturm & Clendon, 2004).

Early classroom communication supports are critical in influencing oral language development and emergent literacy skills. Vygotskian-oriented and social interaction theories (Wertsch, 2004) focused the zone of proximal development that highlighted need for quality interaction between a child and a more knowledgeable adult (like a parent, teacher or speech–language therapist) because it facilitates oral language learning (Kaderavek, 2015). Oral language comprises vocabulary development, awareness of grammar, syntax, knowledge of narrative structures and precursors to comprehension skills (Carroll, Bowyer-Crane, Duff, Hulme, & Snowling, 2011). Furthermore, it is not adult support alone that plays a key role in moving a child through the zone of proximal development but environment as well (Shabani, Mohammad, & Ebadi, 2010).

While oral language development is facilitated in multiple contexts (home, school and social), teachers play a critical role in facilitating literacy skills for academic learning. Language-literacy learning is used in this case because the development of oral language is interlinked with early literacy development in early education settings (Bedore, Pena, Fiestas, & Lugo-Neris, 2020; Moonsamy, 2015; Pelatti, Piasta, Justice, & O’Connell, 2014).

Communication will also be used interchangeably as we refer to the broad scope of language learning, literacy learning and the development of oral and written skills in each area. In South Africa, one of the most significant influences on early language and literacy development, is the reception year (Grade R or kindergarten) that is the entry into the foundation phase of learning (Janse van Rensburg, 2015). Indeed it is the quality of a child’s Grade R year that is a determining factor on how they will develop the language, cognitive and social skills required to build a solid academic foundation (Isaacs, Roberts, Spencer-Smith, & Brink, 2019).

Classrooms supporting effective communication development are critical, particularly for children of low-income and under-served backgrounds who may have limited language-literacy learning opportunities in their home environments (Carolus & Moonsamy, 2019; Dickinson, Hofer, & Rivera, 2019; Neuman, Kaefer, & Pinkham, 2018). Dockrell, Bakopoulou, Law, Spencer and Lindsay (2012) analysed 62 articles and identified three areas of support to facilitate oral language development: (1) language-learning environment (LLE), (2) language-learning opportunities (LLOs) and (3) language-learning interactions (LLIs), which together create communication supporting classrooms (Dockrell et al., 2012).

Teachers are skilled and experienced with implementing the Grade R curriculum, but typically may not receive specific training on how to create communication-supporting classrooms (Justice, Mashburn, Hamre, & Pianta, 2008; Pence, Justice, & Wiggins, 2008). Teachers implement structured aspects of curricula but may be less adept at the process of implementing the conversational dimensions and therefore may benefit from collaborating and being supported by Speech-Language Therapists (SLTs) (Justice et al., 2010). The SLTs have in-depth knowledge around language and communication development and are uniquely equipped to support students, teachers and parents to ensure the achievement of learning outcomes in school (Wium & Louw, 2013). However, SLTs are not experts in classroom teaching. Therefore, SLTs and teachers can partner together to develop equitable primary-level intervention (Pillay & Kathard, 2018) designed to promote healthy classroom communication environments and behaviours for learning (Dockrell et al., 2012), which potentially benefit teachers and all children.

The authors of this article hold the view that teachers have a vast experiential knowledge base about their classrooms and children, and a partnership approach between teachers and SLTs has potential to strengthen communication supporting classrooms.

In South Africa, Grade R is the first point of entry into a 4-year Foundation Phase of learning (Grades R–3) in the school system for most children between 5 and 6 years of age (Department Of Education, White Paper 5, 2001). This is the case for children in low-income marginalised communities who typically do not have access to formal early childhood education between 3 and 5 years (Leseman, 2002). In stark contrast, children in socio-economically advantaged communities have access to quality early learning (PRAESA, 2018). As a means of addressing the inequality in the South African education system, Grade R education was expanded in 2001 in South Africa for the majority, marginalised population.

Despite significant investment in the Grade R infrastructure (Department of Basic Education [DBE], 2013) there are poor outcomes in the quality of education received by learners (Samuels et al., 2015). This situation may be explained by arguing that the educational challenges in South Africa are a consequence of longstanding influences of colonialism and apartheid, which has created a vastly unequal society. In South Africa’s post-1994 democracy, inequality continues to be reflected in an economic area where nearly half of black Africans live below the poverty line compared with 1% with white South Africans (Amnesty International Report, 2020). In the post-1994 context, children’s futures are determined by where they are born (geography), their economic status and skin colour.

Despite the efforts of the Department of Education (DoE), children of mixed race from marginalised backgrounds continue to fall behind their white peers (Johnson, Pesky, & Aulum, 2021; Msila, 2007). Government rural schools in poor communities and urban township schools continue to be impacted by poverty, ill health, poor school infrastructure, limited resources and materials, large numbers of learners per class, limited teacher preparation and didactic instructional techniques (Nel, Mohangi, Krog, & Stephens, 2016; Probyn, 2018).

While the rapid expansion of Grade R learning was a means to address the education inequality gap in South Africa by placing emphasis on basic numeracy, literacy and life skills, there are poor outcomes because of several systemic factors (Samuels et al., 2015).

For this study, two key factors require attention in order to address these inequalities that contribute to poor academic outcomes. Firstly, the need for additional professional development of teachers and secondly, improved alignment between the curriculum and language policy (PRAESA, 2018; Samuels et al., 2015). Although Grade R curricula encourages the development of literacy skills through play-based pedagogy (Mohangi, Krog, Stephens, & Nel, 2016), teachers struggle with practical implementation given that socialisation has occurred in an education system, which typically practices a didactic, transmission mode of teaching (Kathard, Pillay, & Pillay, 2015). Grade R classrooms are commonly attached to primary level schools, implying that teachers are likely to see Grade R as formal learning in preparation for Grade 1. While teachers focus on meeting curriculum goals, they can extend social learning opportunities to facilitate meaningful language and literacy learning (Prinsloo & Bloch, 1999).

Secondly, challenges with the implementation of the language-in-education policy (LiEP) are a key contributing factor to poor academic outcomes (Jordaan, 2011). While the official policy promotes additive bilingualism, in practice, English language learning is actively promoted over African languages as it provides access to the economy (Jordaan, 2011; Probyn, 2018). The LiEP encourages children to be taught in their home languages from Grade R to Grade 3. However, in Grade 4, 80% of children of black African home language backgrounds typically switch to English as the language of learning and teaching. In these cases, they are both taught and assessed in English from there onward. (Heugh, 2013). Classrooms have also become more linguistically diverse, with teachers having limited knowledge and support for how to support language learning, particularly for academic learning (Jordaan, 2011).

The curriculum, however, is premised on the assumption that children have acquired the language and literacy skills to reach curricular goals. The impact of the disjuncture between LiEP and curriculum practices is recognised in Grade 4 when the medium of instruction switches from African home languages to English with no additional language support such as using visuals or allowing students adequate time to process (Kirss, Säälik, Leijen, & Pedaste, 2021).

In a study of teachers’ perspectives of language challenges in intermediate phase classrooms (Grades 4–7) in Western Cape, teachers were concerned that most learners were not meeting grade level outcomes for written language because of multiple systemic challenges as a result of apartheid-related lack of resources, amenities and infrastructure in historically black residential areas (Navsaria, Pascoe, & Kathard, 2011). Teachers in intermediate phase identified the need for early intervention to address language and literacy learning difficulties and the creation of communication supporting classrooms in the foundation phase, beginning in Grade R. The persisting problem of low literacy levels in South Africa strengthens the case for identifying other early supports, which may yield better literacy outcomes (Govender & Hugo, 2020; Spaull, 2015). Against this backdrop, the importance of communication supporting classrooms beginning in preschool (nurseries, crèches, Grade R, etc.) is a critical part of improving learning outcomes.

The large-scale challenge with language-literacy learning in South Africa is becoming a priority for speech–language therapists who are focusing their support on partnerships with teachers, particularly in the Foundation Phase, as a strategy for classroom-based intervention (Kathard & Moonsamy, 2015; Wium & Louw, 2015). This approach of creating meaningful partnerships has the benefit of supporting the teachers to meet the communication learning outcomes required in the National Curriculum and Assessment Policy (DBE, 2021). In addition, it allows SLTs the opportunity to gain a better understanding of classroom dynamics and curriculum.

There is a restrictive therapist-to-population ratio (0.57:10 000) in South Africa (Pillay, Tiwari, Kathard, & Chikte, 2020) with regard to communication professions (speech therapists, speech therapists and audiologists). Therefore, the need to develop innovative population-focused primary level interventions which can support many teachers is vital to ensure the improvement of language-literacy learning (Pillay & Kathard, 2018). However, before interventions are considered, it is important to describe communication supports in Grade R classrooms.

### Communication supporting classrooms

While children’s exposure to language-literacy learning may vary in home environments, the classroom is a structured everyday space to facilitate language-literacy learning. The playground also provides invaluable opportunities for language-literacy learning. Teachers play a key role in in supporting communication by structuring the environment and facilitating interactions and shaping LLOs. Therefore, optimising communication support in a school environment is important.

While there has been a longstanding interest in communication in classrooms, using various assessment methods and tools, for example, the Classroom Assessment Scoring tool (Pianta, La Paro, & Hamre, 2008) and the Classroom Literacy Observation Schedule (Louden, Rohl, Barratt-Pugh, & Brown, 2013) tools have been developed to support research and with a language teaching purpose. However, the Communication Supporting Classroom Observational tool (CSCOT) was developed and validated in the United Kingdom in 2012 for clinical application by speech–language therapists (Dockrell et al., 2012). The CSCOT is structured in three parts:

1. **Language-learning environment**
  The environment refers to the physical classroom environment and how it is organised and managed. It includes observing aspects such as sound levels, provision of learning material such as books and play materials, musical instruments and symbols and pictures to mark the various areas of the class.
2. **Language-learning interaction**
  The LLIs are regarded as the critical aspect of developing oral language as exchanges between teacher and learners is ongoing and provides opportunities for the teacher to be responsive. This interactional support during instruction includes a range of behaviour such as questioning, extending conversations, re-casts (task clarification) and feedback. These interactions are found to facilitate vocabulary development in monolingual speakers and children learning additional languages.
3. **Language-learning opportunities**
  Language-learning opportunities assess the child’s (or children’s) opportunities for language learning and practice. These opportunities include small group work, interactive book readings and structured verbal conversations with adults and peers. These opportunities may be peer–peer or adult–learner dyads.
  The international body of literature confirms that children in contexts of poverty and social disadvantage require communication supporting classrooms as they may not have sufficient environment, stimulation and resources to develop their early communication skills (Ellis & Rowe, 2020; Justice et al., 2010). Environments supporting language-literacy learning are those that are print-rich, have materials and resources, display areas for children’s work and props, which support children’s interactions with each other (Justice, 2004). There are a range of learning environments in South Africa: some have minimal print-based resources, are unhygienic and disorganised; other classrooms are organised, with print-rich environments and a range of learning materials and play resources (Nel, Tlale, Engelbrecht, & Nel, 2016).

Language-learning opportunities are those opportunities that are created in everyday classrooms activities in large or small groups or through individual interaction that provide opportunities, which facilitate language learning.

The activities include play-based activities, storytelling or reading and structured interactions (Guo et al., 2010). The Curriculum and Assessment Policy Statement (CAPS) curriculum encourages a range of oral LLOs, peer conversations, role play, songs and poems (DBE, 2011).

A study of teacher interactions with Grade R children who were refugees (Adams-Obugjele & Moletsane, 2019), found a general lack of quality social interactions with children, as the teacher and assistant were focused on classroom managed activities. Nel et al. (2016) reported that when teachers who were required to provide instruction in English, which is not their home language, they read story books in a structured manner and did not ask questions to engage higher order thinking, hence limiting LLO. These studies recommended teacher professional development to address teachers’ understanding of the CAPS curriculum, to strengthen social interaction skills and facilitate an improvement in the organisation of the learning environment. Teachers are influenced by complex personal and contextual factors, which contribute to how they negotiate changes with instructional processes (Kimathi & Bertram, 2020).

The area of LLI has been the focus of research internationally as the interaction between the teacher and children is critical in language-literacy learning (Swari, Tantra, & Pratiwi, 2020). Rimm-Kaufman et al. (2002). observed variation in activities in preschool classrooms environments across three states in the United States of America, with the most frequent activity being structured teacher-directed activity and whole group instruction. While more informal and play-based learning occurs in preschool classrooms, the early childhood classroom is teacher-led, with the teacher contributing to 93% of directive speech acts. This is for varied purposes including confirming ordering, explaining, requesting and telling (Swari et al., 2020). In this same environment, children used speech acts mainly for the purpose of telling when prompted or initiated by a teacher (Early, Maxwell, Ponder, & Pan, 2016).

Wasik, Bond, and Hindman (2006) reported that when teachers were supported in their delivery of literacy intervention, they made positive changes in their interactions during book reading and other activities and practiced active listening, extended conversations, asked questions and provided feedback. Children’s language development also benefitted through these interactions.

In light of the death of research on Grade R communication supporting classrooms, this study set out to describe LLE, LLO and LLI Grade R classrooms attached to public schools in the Western Cape, South Africa. This knowledge will serve to inform SLT practices in supporting Grade R teachers.

## Methodology

### Aims and objectives

The primary aim of the study was to describe how Grade R classrooms scored on the domains of CSCOT tool, namely:

1. language-learning environments
2. language-learning interactions
3. language-learning opportunities.

The secondary aim of the study is to discuss the implications for speech–language therapy practice within the education or schooling system and as partners with teachers.

### Methodology

#### Research design

The study used a cross-sectional descriptive survey observational design whereby classrooms were observed using a criterion referenced tool – the CSCOT (Fraenkel & Wallen, 2000). The design was suited to collecting data from a large number of classrooms. The classroom and its teacher(s) and learners was the unit of data collection and analysis.

#### Criteria for inclusion of classrooms

The following criteria were used for including classrooms in the study:

- public sector school following the national Grade R curriculum
- classroom with a teacher who routinely taught the Grade R class (i.e. no substitute teachers or locum teachers).

#### Criteria for exclusion

Classrooms attached to independent schools and early childhood development centres were excluded.

#### Sampling

Stratified adaptive cluster sampling was used to ensure a representative sample of schools in urban and rural or remote areas, as well classrooms from schools across the academic performance range, that is, higher and lower performance range. This sampling strategy allowed the schools in both rural and urban areas in the Western Cape equal opportunity for participation. Within the schools selected, a cluster of Grade R classrooms were selected.

#### Recruitment

Researchers first received permission from the Western Cape Education Department to conduct the study. Thereafter, school principals were contacted to obtain their permission to conduct the study. After information was provided and it was established that the classrooms met the criteria for inclusion, arrangements were made for data collection to commence with teachers who consented to participate.

### Method

A structured observation method was employed, with the CSCOT used as the observational tool. Prior to the study, research assistants were trained on conducting observations using the tool until they established a good inter-rater reliability (interclass correlation of 0.9). The research assistants chosen were students at the University of Cape Town in the field of linguistics, communications and other disciplines with a basic knowledge of communication.

A total of 136 classrooms were observed for 1 h per classroom. Where feasible, two raters were available per classroom, meaning that many classrooms had more than one observation. The total number of observations were 223, with 87 classrooms having multiple observations per classroom. Each rater observed made independent observations. The observation occurred in the morning session of the school routine, which was typically a literacy or numeracy learning activity. The data were collated and entered into a spreadsheet, checked and verified by an independent researcher.

### Research instrument communication supporting classroom observational tool

The authors chose the CSCOT because it is a tool developed specifically to support SLT practices in the early years – from Grade R to Grade 2 (Dockrell et al., 2012). It was created to profile learning spaces or classroom environments and explore the various dimensions that supported the development of language skills (Dockrell et al., 2012). Furthermore, the South African school systems are similar in ethos, spirit and function to British educational contexts, sharing similar practices, that is, classrooms and education have a longstanding influence from British education system, which therefore makes the tool a good starting point to describe the South African classroom environment.

Furthermore, as part of an undergraduate research project that was conducted at the University of Cape Town (Harty et al., 2013), the CSCOT was examined by a panel of experts to determine the face and content validity of the tool’s relevance and suitability in a South African context (Harty et al., 2013). Finally, the CSCOT used a comprehensive approach by examining the physical environment (LLE – 18 items), LLI (19 items) and LLO (5 items) as the domains of the tool. For LLE, the observer noted if the item was present or not. For LLI and LLO, the observer marked the number of times the behaviour was observed in the hour of observations – from 0 to a maximum of 5 observations.

### Procedure

The data were collected as two parts – one research team focused on the rural or remote schools while the other team collected data in urban areas. The teams arranged the daily schedules for observations. Where feasible, two researchers entered a classroom and observed the classroom for an hour, usually during a morning routine followed by a language or mathematics lesson. Each rater completed the CSCOT independently and the team head collected the hard copies of the data. The data per classroom observation was then captured independently by a researcher onto an Excel spreadsheet. For each observation, the data were captured per item for each area of the CSCOT and were identified. The data entries were then verified by a research assistant.

### Data analysis

Descriptive tables were produced to show the frequency of occurrence for each item in LLE, LLI and LLO. Scoring for LLE, LLI and LLO items followed the same procedure suggested by the developer (Dockrell et al., 2015). For LLE items, a score of 1 was assigned for each item if the item was present and 0 if the item was absent. For LLI items, scores were assigned on an ordinal scale of 5, with a score of 0 indicating the absence of an item, while a score from 1 to 5 indicates increasing presence of an item in the classroom. Language-learning opportunities items followed the same scoring scheme as LLI. For LLE, the frequency and percentage that specific items scored a ‘1’ for each item was recorded in Table 1.

**TABLE 1:** Number of observations that indicated the presence of each item in the language-learning environment area (*N* = 223).

LLE item	Frequency	%
11. There is good light.	222	99.6
1. The classroom is organised to emphasise open space.	218	97.8
13. Resources that are available for free play are easily reached by the children or easily within their line of vision.	214	96.0
6. Some classroom displays include items that invite comments from children.	211	94.6
10. Transition times are managed effectively, so that noise levels are not excessive, and children know what to expect next.	210	94.2
9. Background noise levels are managed consistently throughout the observation and children and adults are able to hear one another with ease.	199	89.2
12. The majority of learning resources and materials are labelled with pictures or words.	196	87.9
7. Book-specific areas are available.	191	85.7
5. Children’s own work is displayed and labelled appropriately.	173	77.6
14. An appropriate range of books is available in the book area (e.g. traditional stories, bilingual or dual language books and a variety of genres and books related to children’s own experiences).	145	65.0
2. Learning areas are clearly defined throughout the classroom.	135	60.5
3. Learning areas are clearly labelled with pictures or words throughout the classroom.	111	49.8
4. There is space for privacy or quiet areas where children can retreat to have ‘downtime’ or engage in smaller group activities. These areas are less visually distracting.	88	39.5
17. Good quality toys, small world objects and real or natural resources are available.	58	26.0
19. Role play area is available (shopping, dress up, building designated area).	52	23.3
8. Literacy specific areas are available (writing, reading activities, colouring, etc.).	35	15.7
15. Nonfiction books, books on specific topics or interests of the children are also available in other learning areas.	32	14.3
18. Musical instruments and noise makers are available.	17	7.6
16. Outdoor play includes imaginative role play (constructive language display).	9	4.0

LLE, language-learning environment.

For LLI items, the frequency and percentage of individual observations that scored ≥ 3 were recorded in Table 2. For LLO items, the frequency and percentage of individual observations that scored ≤ 1 were recorded; this decision was made post hoc after examining the distribution of scores in the LLO domain.

**TABLE 2:** Number and proportion of observations that indicated a score ≥ 4 in each item of the language-learning interaction area (*N* = 223).

LLI item	Frequency	%
1. Adults use children’s names, draw attention of children.	211	94.6
6. Pausing: Adult pauses expectantly and frequently during interactions with children to encourage their turn-taking and active participation.	163	73.1
5. Pacing: Adult uses a slow pace during conversation; give children plenty of time to respond and take turns in interacting with them.	161	72.2
18. Turn-taking is encouraged.	126	56.5
8. Imitating: Adult imitates and repeats what child says more or less exactly.	92	41.3
4. Adults use symbols, pictures and props (real objects) to reinforce language.	86	38.6
13. Open questioning: Adult asks open-ended questions that extend children’s thinking (what, where, when, how and why questions).	81	36.3
11. Labelling: Adult provides the labels for familiar and unfamiliar actions, objects or abstractions (e.g. feelings).	54	24.2
3. Natural gestures (action to support what is being said ‘pop’) and some key word signing are used in interactions with children.	51	22.9
7. Confirming: Adult responds to the majority of child utterances by confirming understanding of the child’s intentions. Adult does not ignore child’s communicative bids. (‘Yeah, mm, yes, OK, really’)	36	16.1
9. Commenting: Adult comments on what is happening or what children are doing at that time.	30	13.5
10. Extending: Adult repeats what child says and adds a small amount of syntactic or semantic information.	18	8.1
17. Adult models language that the children are not producing yet.	13	5.8
16. Adult uses contrasts that highlight differences in lexical items and in syntactic structures (opposites: big, small and plurals and verbs: -ed, -es).	10	4.5
2. Adults move down to the child’s level when interacting with them.	9	4.0
12. Adult encourages children to use new words (what are the new words?) in their own talking.	7	3.1
19. Children’s listening skills are praised.	2	0.9
20. Children’s nonverbal communication is praised.	1	0.4
14. Scripting: Adult provides a routine to the child for representing an activity (e.g. ‘Firstly, you go up to the counter. Then you say, “I want milk…”’) and engages the child in known routines (e.g. ‘Now it is time for circle time. What do we do first?’).	0	0.0
15. Adult provides children with choices (e.g. ‘would you like to read a story or play on the computer?’).	0	0.0

LLI, language-learning interaction.

### Validity and reliability

The CSCOT was deemed to be reliable and valid (Dockrell, Bakopoulou, Law, Spencer, & Lindsay, 2015) through extensive testing in United Kingdom classrooms and further assessment for applicability in South Africa (Harty et al., 2013). Furthermore, the reliability of the observations was strengthened by training of six research assistants who participated in three weekly training sessions. In these sessions, rater responses were compared with expert observations and this process was repeated until the researchers were satisfied with raters’ progress in training. Then raters were required to watch an hour-long video of classroom interactions in English, Afrikaans and Xhosa classrooms and complete the tool. These results scores were used to determine inter-rater reliability, using the intraclass correlation coefficient (ICC). For the English classroom the ICC was 0.99; for the Afrikaans classroom 0.93 and for the isiXhosa classroom the ICC was 0.91, which indicates a strong level of agreement between raters. The ICC values indicated that language matching was not a necessity when observing a classroom. However, feedback from raters indicated that there was greater ease when they observed classrooms in a language that they were competent in. Therefore, there was at least one rater per classroom who was competent in the language used in the classroom for instructional purposes.

**TABLE 3:** Number and proportion of observations that indicated a score ≥ 3 in each item of the language-learning interaction area (*N* = 223).

LLI item	Frequency	%
1. Adults use children’s names, draw attention of children.	217	97.3
5. Pacing: Adult uses a slow pace during conversation; give children plenty of time to respond and take turns in interacting with them.	186	83.4
6. Pausing: Adult pauses expectantly and frequently during interactions with children to encourage their turn-taking and active participation.	186	83.4
18. Turn-taking is encouraged.	160	71.7
4. Adults use symbols, pictures and props (real objects) to reinforce language.	125	56.1
8. Imitating: Adult imitates and repeats what child says more or less exactly.	122	54.7
13. Open questioning: Adult asks open-ended questions that extend children’s thinking (what, where, when, how and why questions).	112	50.2
3. Natural gestures (action to support what is being said ‘pop’) and some key word signing are used in interactions with children.	87	39.0
11. Labelling: Adult provides the labels for familiar and unfamiliar actions, objects or abstractions (e.g. feelings).	73	32.7
7. Confirming: Adult responds to the majority of child utterances by confirming understanding of the child’s intentions. Adult does not ignore child’s communicative bids. (‘Yeah, mm, yes, OK, really’)	55	24.7
9. Commenting: Adult comments on what is happening or what children are doing at that time.	55	24.7
2. Adults get down to the child’s level when interacting with them.	35	15.7
10. Extending: Adult repeats what child says and adds a small amount of syntactic or semantic information.	30	13.5
17. Adult models language that the children are not producing yet.	29	13.0
16. Adult uses contrasts that highlight differences in lexical items and in syntactic structures (Opposites: big, small and plurals and verbs: -ed, -es).	28	12.6
12. Adult encourages children to use new words (what are the new words?) in their own talking.	9	4.0
14. Scripting: Adult provides a routine to the child for representing an activity (e.g. ‘First, you go up to the counter. Then you say, “I want milk…”’) and engages the child in known routines (e.g. ‘Now it is time for circle time. What do we do first?’).	4	1.8
19. Children’s listening skills are praised.	2	0.9
20. Children’s nonverbal communication is praised.	2	0.9
15. Adult provides children with choices (e.g. ‘Would you like to read a story or play on the computer?’).	0	0.0

This is from the Communication Supporting Classrooms Observational Tool (Dockrell et al., 2012).

LLI, language-learning interaction.

**TABLE 4:** Number and proportion of observations that indicated a score ≤ 1 in each item of the language-learning opportunity area (*N* = 223).

LLO item	Frequency	%
Attempts are made to actively include all children in small group activities	220	98.7
Children have opportunities to engage in interactive book reading facilitated by an adult (e.g. asking predictive questions, joining in with repetitions, story packs etc.).	217	97.3
Small group (three or more kids) work facilitated by an adult takes place.	212	95.1
Children have opportunities to engage in structured (at least three turns) conversations with peers (talking partners).	211	94.6
Children have opportunities to engage in structured conversations with teachers and other adults.	145	65.0

This is from the Communication Supporting Classrooms Observational Tool (Dockrell et al., 2012).

LLO, language-learning opportunities.

### Ethical considerations

The studies in the urban and rural contexts were approved by the University of Cape Town Faculty of Health Sciences Research Ethics Committee (HREC) (reference number: HREC 481/2014; 480/2014).

### Results

**FIGURE 1:**
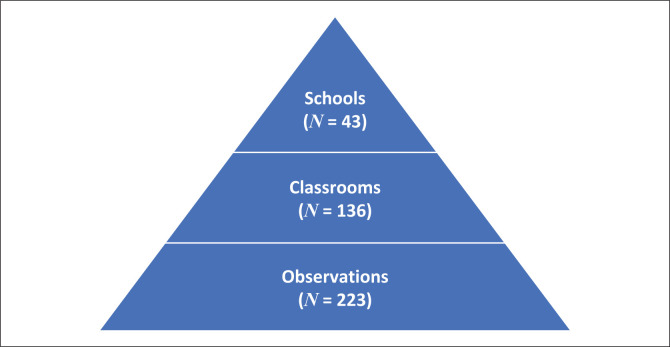
Number of schools, classroom and observations included.

Data from 136 classrooms with 223 observations were analysed and included schools in rural or remote and urban areas and across the academic performance range. The observations were conducted on 136 classrooms drawn from 43 schools (see Figure 1).

The teachers were all female, with an average age of 39.95 (s.d. = 11.2) years. The class size averaged 28 learners (s.d. = 6.7). The language of instruction across the Western Cape varied across classrooms where English (35%) and Afrikaans (39.7%) were mainly spoken in urban areas, whereas isiXhosa (7.4%) was mainly spoken in rural areas. The learners were of diverse language backgrounds and had multilingual exposure in English, Afrikaans and isiXhosa. Teachers and learners shared a common language in 99 classrooms. In the other 37 classrooms, this was not the case, implying that children were learning in a language that was different from their home language (whether it be isiXhosa, French or an additional African language). All teachers had formal training in education, and 86.8% teachers demonstrated additional training or qualification for teaching Grade R children. On average, they had 8.86 years (s.d. = 8.6) of experience in Grade R teaching specifically.

Overall, the items that were most commonly occurring were numbers 1 (adults use children’s names, draw attention of children), 5 (Pausing: Adult pauses expectantly and frequently during interactions with children to encourage their turn-taking and active participation) and 6 (Pacing: Adult uses a slow pace during conversation; give children plenty of time to respond and take turns in interacting with them) with a large majority of classrooms achieving scores for these items.

The item least commonly occurring was item 14 (Scripting: Adult provides a routine to the child for representing an activity) with no classrooms demonstrating evidence of this strategy. This indicates that while teachers are using basic communication practices, the finer aspects of language that encourage children to practice language in interactions are missing in classrooms. This may indicate that teaching styles in most classrooms remain didactic.

### Objective 1: Language-learning environment

**FIGURE 2:**
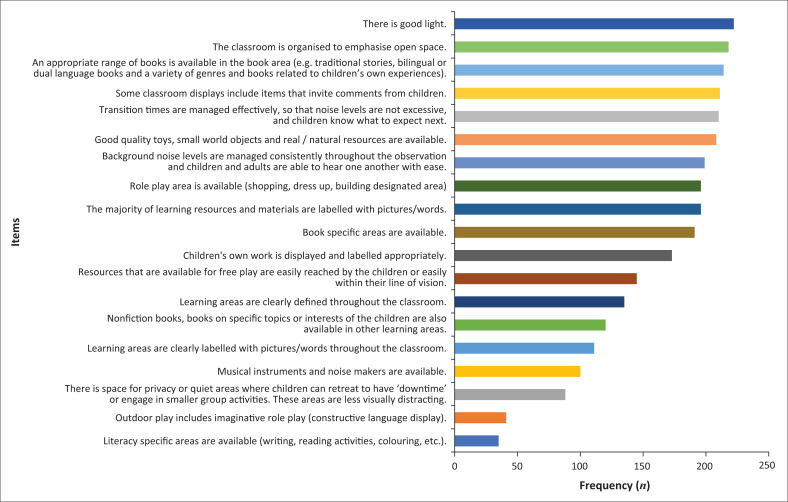
Frequency of items observed – Language-learning environment.

The items occurring frequently (75% and greater) across classrooms indicated that the classrooms had adequate lighting, books and quality toys and natural resources for learning (e.g. shells, pebbles, pinecones). There were designated book and play areas with materials labelled with pictures or words. Classrooms had displays that invited comments from children. The ambient sound levels were low while children’s work was labelled and displayed clearly. Less frequently observed items (65% and less) were resources for free play and availability of nonfiction books. Items that were observed 50% or fewer times included learning areas, which were clearly defined and labelled throughout the class, availability of music instruments and noisemakers and privacy spaces for downtime. There was minimal observation (less than 20%) of outdoor play, imaginary (role) play areas and literacy-specific areas (see figure 2).

### Objective 2: Language-learning interactions

**FIGURE 3:**
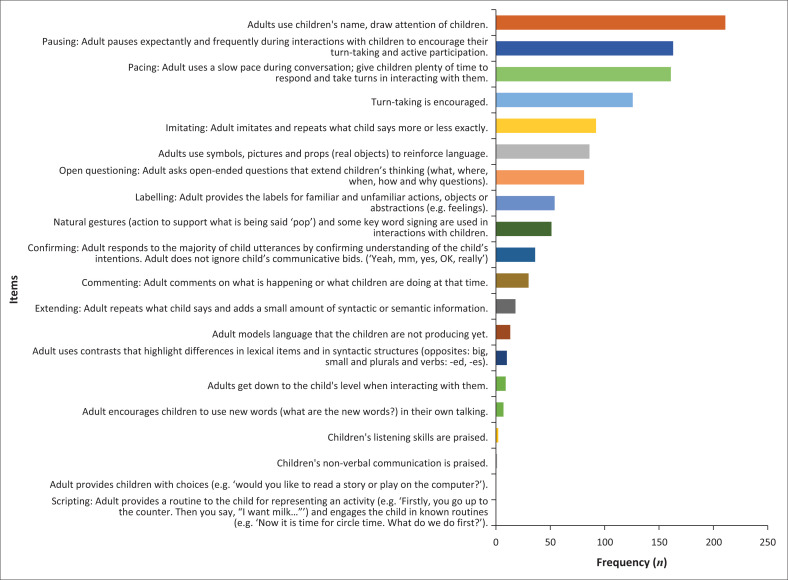
Frequency of items observed – Language-learning interactions (> 4).

Note that scores of 4 and 5 are considered clinically relevant as ‘good’ achievement in an LLI and LLO domain. This result indicates the number or percentage of observations, which had a score of 4 or greater than 4, meaning that these were well-established or commonly occurring (see figure 3).

The commonly occurring item was that the teacher used the child’s name (in 94.62% of classrooms), pausing and pacing while turn-taking in 56.5% of classrooms. Less commonly occurring behaviours were teachers’ use of symbols, open questioning, labelling natural gestures and confirming. Behaviours observed infrequently were commenting, extending, using adult models that the child is not using yet; adults use of contrasts, encouraging children to use new words, praising listening skills and nonverbal communication. There were no observations of scripting and choices for children. Over 10 items on this scale did not occur commonly across classrooms, for example, praising of nonverbal communication, praising listening skills and encouraging children to use new words.

As a less stringent criteria of a frequency of occurrence of the items 3 or more times indicated a similar pattern of analysis as 4 or more observations. Sometimes the more complex interaction behaviours are observed across classrooms. These items relate to communication strategies used by teachers to scaffold the language learning of children and could be targeted in teacher training by SLTs.

### Objective 3: Language-learning opportunities

**FIGURE 4:**
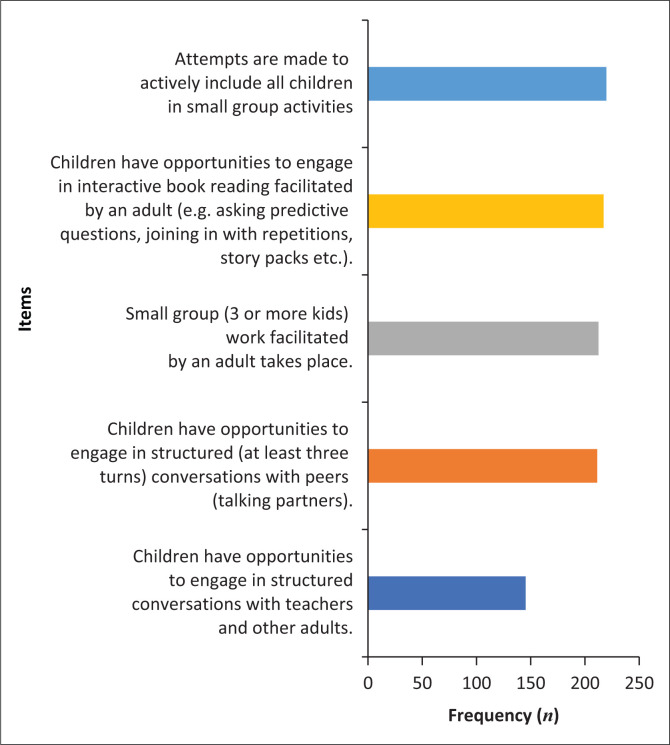
Frequency of items observed – Language-learning opportunities (scores ≤ 1).

This area of 5 items indicates that in general there were few observations of LLO. These included attempts to include children in small group activity; opportunities to engage in interactive book reading facilitated by an adult; small group work facilitated by adults; opportunities to take three turns; and structured opportunities for developing conversations (see figure 4).

## Discussion

Overall results indicate that the LLE was more robust than the LLI and LLO domains. This result indicates that while the environment had material resources to support communication in classrooms, the frequency of behaviours for LLI and LLO could be extended. Furthermore, relative to the scoring and assumptions of the CSCOT, the results indicated that although there was basic equipment and structure, there were still a number of items which were not commonly observed in the classrooms in this study. Clearly, contextual and resource issues continue to play a role in creating optimal environments for language-literacy learning. For example, many classrooms in rural areas (previous mixed race or black areas under apartheid) were created out of shipping containers, which placed resource and space constraints on the class. Moreover, many schools in the rural areas had minimal access to additional resources such as musical instruments, books in many languages and a range of types of books and outdoor play areas. For many children in marginalised communities, outdoor play is restricted because of lack of resource and community safety concerns.

Globally, the expansion of physical environment to include creative, outdoor and free play is regarded as critical to early learning (Shaari & Ahmad, 2015). Therefore, this is an important area to consider when looking to improve language learning. While there are aspects of LLI that build engagement with learners, the less frequently observed were skills related to giving children time to engage in conversations with peers and other adults in the classroom. This observation indicates that opportunities for verbal interaction are restricted. It is likely that the teacher is structuring the classroom, relative to CAPS curriculum and getting children ‘school-ready’.

Didactic teaching methods and classroom structures observed in many traditional classroom set-ups may explain the less frequent interactive behaviours (Kariippanon, Cliff, Lancaster, & Okely, 2019). The observations of the researchers were that there was a formal delivery of a structured routine which teachers follow that may explain the common restrictive patterns across classrooms. For classrooms situated in contexts of social disadvantage in which children may not have access to enriched LLOs, it is essential that the teacher is supported – particularly when learning is occurring mediated in a second or additional language (Jordaan, 2011; Moonsamy & Kathard, 2015). Creating more interactive scaffolded conversations and LLOs are the main focus areas for supporting communication development is a key primary level support.

## Implications

### Building collaborative partnerships

It should be noticed that the observations in this study were an etic or outsider view (from research observations) on what happens in the classroom using the CSCOT. While this study provides insights into classroom environments, it is acknowledged that the next step in the research process is to develop a deeper level of understanding that can be gained by further teacher–SLT engagement to gain a more robust, contextualised understanding of classroom language-literacy learning culture prior to any intervention. This collaboration will serve to understand the teachers’ everyday context and constraints and to identify the key intervention priorities. The CSCOT was developed with intention to support teachers in self-assessment of their classroom communication supports and Law, Tulip, Stringer, Cockerill, and Dockrell (2019) found that teachers across Reception Class, Year 1 and 2 in north-east England found the tool easy to use and found LLO helpful as it related to everyday classroom language learning, while LLI was found to be helpful in scaffolding interactive language learning and thinking.

The potential benefits and strategies for engaging with teachers to develop communication supporting environments are gaining momentum internationally. Hamre, Hatfield, Pianta, and Jamil (2014) confirmed that interventions with teachers add value as it facilitates responsive teaching and an emotionally sensitive environment. They provide various creative options for supporting teachers (Hamre et al., 2014). These included in-class support and online support.

A further study also confirmed that carefully planned teacher professional development interventions could have positive effects (Early et al., 2016). Justice et al. (2008) demonstrated the value of two things, namely the Read it Again (RIA) strategy with preschool teachers supporting language-literacy learning and speech–language therapists providing curriculum-based supports. SLTs have a collaborative role to play in supporting teachers to create meaningful interactions in their classrooms and these interactions have a positive effect on several child communication outcomes. Piasta, Justice, McGinty, and Kaderavek (2012) similarly demonstrated that preschool teachers benefit from support with conversational strategies but required more intensive support to the development of language developing strategies. Piasta et al. (2020) in a randomised controlled trial study with 109 teachers and 726 children (children with disabilities and their peers) using the RIA shared book reading intervention found that there was a significant increase in teachers’ provision of explicit instruction in print knowledge, vocabulary, phonological awareness and narrative. The study once again confirms that partnerships with teachers can support and change communication behaviour.

### Expanding service delivery approaches

The SLTs in South Africa are considering how to serve population needs to deliver equitable services through expanding their service delivery approach from the traditional individual pullout model. Initial evidence from small scale studies in South Africa, Wium, Louw and Eloff (2011) confirmed the value of working with teachers in supporting literacy learning in foundation phase. In a small-scale study, Carolus and Moonsamy (2019) demonstrated that collaboration between preschool teachers and SLTs highlighted the positive benefits as teachers changed their explicit instructional behaviour to support emergent literacy.

The benefits of collaborative practices between teachers and SLTs were also found in a study which used environmental print to support emergent literacy in under-served communities (Giacovazzi, Moonsamy, & Mophosho, 2021). All children, including children with specific communication disabilities stand to benefit from interventions that are teacher-led and can be practised on an everyday basis. Importantly, the collaborative approach values the teacher as a professional with agency who has a close understanding of the context and therefore can decide how to implement newly learned behaviours.

In South Africa, the language-literacy crisis calls for the urgent scaling-up of primary level supports. To create such supports, SLTs must be politically conscious, viz. to address equality, equity and social justice as tools to transform practice (Kathard & Pillay, 2013), and skilled in advocating for expanding their practices in the education system from the traditional pull-out models and small group work. Furthermore, they must be skilled to work in collaborative partnerships with teachers. The coronavirus disease 2019 (COVID-19) pandemic has created opportunities for considering innovations in service delivery approach. A further avenue of support is for SLTs to work with the preservice teacher education programme.

### Synergising communication supporting classrooms and curriculum goals

The SLTs are more likely to find synergy with teachers when they make explicit the importance of communication support, which remains invisible, yet critical. Tools such as the CSCOT make LLE, LLI and LLO explicit and contribute to strengthening the curriculum in two ways. Firstly, by supporting teachers in how to create a communication supporting classroom to reach their literacy goals. Secondly, supporting teachers with how to embed communication skills to strengthen their pedagogy. One avenue currently being pursued in South Africa is the use of play-based pedagogy in Grade R. While play supports children’s learning (Miller, 2000), Aronstam and Braund (2016) found that Grade R teachers viewed play as a recreational activity, which is not beneficial to learning and meaningful interaction. They concluded that it was important to improve on the quality of play in Grade R through facilitating teachers critical understanding of play as a pedagogy.

Harty, Alant and Uys (2007) demonstrated the utilisation of play-based activity in aided language stimulation (AiLS) in South Africa. They implemented a two-phase training programme and coached six grade R teachers to improve interaction in the classroom using AiLS facilitator boards. Data indicated that teachers improved in the areas of classroom management, AiLS strategy use and teacher interactions after a 6-week training programme.

## Strengths and limitations

The CSCOT was an appropriate tool to use given the exploratory nature of the research. It was simple and easy to use. It also provided training to other researchers with no background in education a fairly straightforward process. The comments area on the tool allowed researchers to make additional observations in each area, which allowed for in depth analysis. One notable shortcoming of the CSCOT is that there was little to compare the results to because of how new it was to the South African context. There is also no overall score that would allow researchers to give classrooms one score to represent the quality of the communication environment and subsequently use the scores of each area to indicate areas of strengths and weaknesses.

Furthermore, the area of LLO was limited as a scale with few items with which to measure opportunities children have to practise language. As external observers, there was great opportunity to build relationships with teachers. They were receptive to having us in their classrooms and very willing to open up about the celebrations and challenges of their everyday life.

## Conclusion

While the LLE in Grade R classrooms were higher than the LLO and LLI scores, there is opportunity for improvement in all areas. The very low score on LLO reflect the minimal LLO provided in the period observed. Similarly, critical LLI items that support children’s language-literacy learning was observed infrequently.

The study raises the awareness of the importance, and yet the invisibility, of the types of behaviours, which create communication supporting classrooms – everyday environments and exchanges that can potentially strengthen language-literacy learning. We argue for collaborative research and implementation practice partnerships between teachers and SLTs to create primary level supports to contribute to improving learning outcomes for children in Grade R.
